# Integrated Assessment of Nickel Electroplating Industrial Wastewater Effluent as a Renewable Resource of Irrigation Water Using a Hydroponic Cultivation System

**DOI:** 10.3389/fpls.2021.609396

**Published:** 2021-02-23

**Authors:** Y. N. Chow, L. K. Lee, N. A. Zakaria, K. Y. Foo

**Affiliations:** ^1^River Engineering and Urban Drainage Research Centre (REDAC), Universiti Sains Malaysia, Nibong Tebal, Malaysia; ^2^School of Industrial Technology, Universiti Sains Malaysia, Gelugor, Malaysia

**Keywords:** closed hydroponic, food safety, nickel electroplating industry, nutrient recycling, phytotoxicity, wastewater reuse

## Abstract

Nickel, a micronutrient essential for plant growth and development, has been recognized as a metallic pollutant in wastewater. The concentration of nickel ions in the water course, exceeding the maximum tolerable limit, has called for an alarming attention, due to the bioaccumulative entry in the water–plant–human food chain, leaving a burden of deteriorative effects on visible characteristics, physiological processes, and oxidative stress response in plants. In this work, the renewable utilization of nickel electroplating industrial wastewater effluent (0, 5, 10, 25, 50, and 100%) as a viable source of irrigation water was evaluated using a hydroponic cultivation system, by adopting *Lablab purpureus* and *Brassica chinensis* as the plant models, in relation to the physical growth, physiological and morphological characteristics, photosynthetic pigments, proline, and oxidative responses. The elongation of roots and shoots in *L. purpureus* and *B. chinensis* was significantly inhibited beyond 25 and 5% of industrial wastewater. The chlorophyll-*a*, chlorophyll-*b*, total chlorophyll, and carotenoid contents, accompanied by alterations in the morphologies of xylem, phloem, and distortion of stomata, were recorded in the industrial wastewater-irrigated groups, with pronounced toxicity effects detected in *B. chinensis*. Excessive proline accumulation was recorded in the treated plant models. Ascorbate peroxidase (APX), guaiacol peroxidase (POD), and catalase (CAT) scavenging activities were drastically altered, with a profound upregulation effect in the POD activity in *L. purpureus* and both POD and APX in *B. chinensis*, predicting the nickel-induced oxidative stress. Conclusively, the diluted industrial wastewater effluent up to the optimum concentrations of 5 and 25%, respectively, could be feasibly reused as a renewable resource for *B. chinensis* and *L. purpureus* irrigation, verified by the minimal or negligible phytotoxic implications in the plant models. The current findings have shed light on the interruption of nickel-contaminated industrial wastewater effluent irrigation practice on the physical and biochemical features of food crops and highlighted the possibility of nutrient recycling via wastewater reuse in a sustainable soilless cultivation.

## Introduction

Phytotoxicity of heavy metals, mainly featured by the alterations of numerous physiological processes at the molecular or cellular level, including the inactivation of enzymes, blockage of functional groups at the metabolically important molecules, substitution or displacement of essential elements, and disruption of membrane integrity, has emerged to be a worldwide agenda among the scientific community (Khan et al., 2020; Rizvi et al., 2020; Wakeel et al., 2020). The bio-accumulative entry and indiscriminate discharge of these heavy metals, in particular nickel, lead, and chromium, to the waterways, soils, and eventually to the food chain, specifically from the anthropogenic activities of metal mining, smelting, and electroplating industries; fossil fuel burning; steel manufacture; emissions from vehicle; disposal of household, industrial, and municipal waste; fertilizer and organic manure application; and other miscellaneous sources, constitute a sharp and alarming health risk to the public health and ecosystems (Ceasar et al., 2020; Yan et al., 2020). This pollution becomes more drastic, specifically in urban and agricultural catchments, which receive a variety of organic manures heavily loaded with different toxic metals. In such cases, both the treated and untreated municipal wastewaters in the vicinity of large cities, is a major utility widely applied for the irrigation practice.

The presence of elevated levels of heavy metals in the growing medium of germinating seeds has been reported to suppress the translocation and mobilization of reserve nutrients from the reserve tissues to the growing regions. Similarly, buildup of heavy metals in the rooting medium would significantly retard plant growth; elicit perturbations in cellular metabolism; affect the uptake of potassium, calcium, and magnesium; and decrease the number of flowers, fruits, and crop yield. The most common toxicity symptoms in plants are necrosis, chlorosis, wilting, and disturbance of physiological processes, including photosynthesis, transport of photo-assimilates, mineral nutrition, and plant structure damage (Kasprzak et al., 2003; Rather et al., 2020).

Among all, nickel toxicity has received aesthetic concern, owing to its excessive wide-scale applications in different industries. Under stress conditions, including exposure to the excess concentrations of heavy metals, an imbalanced generation and degradation of reactive oxygen species (ROS) could arise in plant tissues (Georgiadou et al., 2018). This may subsequently result in oxidative injuries of a wide range of important macromolecules including proteins, lipids, and nucleic acids. During this stage, these cells are protected against free oxyradicals by the enzymic and non-enzymic systems that serve as free radical scavengers. The major enzymatic detoxifiers of hydrogen peroxide (H_2_O_2_) in plants are ascorbate peroxidase (APX, EC 1.11.1.11) and catalase (CAT, EC 1.11.1.6). CAT would catalyze the detoxification of H_2_O_2_ into water and oxygen molecules; alternatively, H_2_O_2_ could be eliminated via the ascorbate/glutathione reaction cycle by APX (Kapoor et al., 2019).

Similarly, the rising peroxidase activity is known as a physiological response to abiotic and biotic stressors. Peroxidases are primarily detected in endoplasmic reticulum, vacuoles, cell walls, and Golgi apparatus in plants, and the distribution is presumably associated with different physiological functions. This enzyme has been hypothesized to be a potential bioindicator for sublethal metal toxicity in plants. The accumulation of proline, one of the most widespread proteinogenic metabolites originating from plant tissues against stress conditions, has been well documented (Du et al., 2020). The protective roles of proline have been attributed to its ability to react as an osmoprotectant, source of carbon and nitrogen, membrane stabilizer, protectant of enzymes, and ROS scavenger (Chandrasekhar and Ray, 2017). Meanwhile, an excess of metallic ions has demonstrated destructive effects on the functionality and content of the photosynthetic pigmentations, usually governed by the inhibition of pigment biosynthesis, formation of metal-substituted chlorophylls, or direct oxidative damages on the pigments. This study was conducted to gear toward an in-depth understanding of the concept of renewable utilization of nickel electroplating industrial wastewater effluent, with respect to the physical, physiological, biochemical, and morphological responses of *Lablab purpureus* and *Brassica chinensis* using a closed-hydroponic system. The time course of changes in the (a) photosynthetic pigments, (b) scavenging activities of antioxidative enzymes, and (c) proline accumulation in relation to the wastewater effluent was analyzed. In parallel, the representative growth parameters, including the physical response and the morphological alterations of shoots, leaves, and roots, were elucidated.

## Materials and Methods

### Reagents and Chemicals

The required reagents and chemicals were of analytical grade and purchased from Merck (Darmstadt, Germany): acetone, ascorbic acid, ethylenediaminetetraacetic acid (EDTA), bovine serum albumin, methanol, Coomassie Blue dye G250, aqueous sulfosalicylic acid, H_2_O_2_, proline, glacial acetic acid, nitric acid, and toluene. On the contrary, sodium hypochlorite, guaiacol, polyvinylpyrrolidone (PVP), ninhydrin acid, and sodium phosphate buffer were supplied by Sigma-Aldrich (St. Louis, MO, United States).

### Nickel Electroplating Industrial Wastewater Effluent

The wastewater sample was collected from the effluent discharge point at the multi-electroplating industrial zone of Bukit Minyak, Penang, Malaysia. Water sampling was carried out using 500-ml polyethylene bottles, which were pretreated with concentrated HNO_3_ for sample preservation to minimize heavy metals degradation by microorganisms. The physicochemical properties, with respect to the pH, electrical conductivity (EC), total suspended solid (TSS), total dissolved solid (TDS), total phosphorus (TP), ammoniacal nitrogen (AN), dissolved oxygen (DO), 5-day biochemical oxygen demand (BOD_5_), and chemical oxygen demand (COD), were determined *in situ* or using a spectrophotometer, by adopting the American Public Health Association (APHA) standard procedures (HACH, DR3900, United States) (American Public Health Association [APHA], 1995). The concentrations of heavy metals were measured with an inductively coupled plasma mass spectrometry (ICP-MS, NexION 300, PerkinElmer, United States). As an indicator of irrigation viability, the pH, EC, TSS, TDS, COD, BOD_5_, TP, AN, and DO were analyzed. The pH, TDS, and BOD_5_ of the industrial wastewater effluent fell within the suitable range for the applications of wastewater reuse (World Health Organization [WHO], 2006). The raw industrial wastewater effluent, diluted at 0 (control), 5, 10, 25, 50, and 100%, was applied as irrigation water (Table 1).

**TABLE 1:** Physicochemical characteristics of the nickel electroplating industrial wastewater effluent.

Parameter	Unit	Nickel electroplating industrial wastewater effluent	Recommended maximum level for irrigation (World Health Organization [WHO], 2006)
pH	–	6.5 ± 0.4	6.5–8.0
EC	dS/m	1.50 ± 0.07	0.70–3.00
TSS	mg/L	30 ± 1.43	N.A.
TDS	mg/L	558 ± 20.52	500–2,000
COD	mg/L	260 ± 10.12	N.A.
BOD_5_	mg/L	20 ± 0.94	10–30
DO	mg/L	2.20 ± 0.98	N.A.
Turbidity	NTU	14 ± 0.50	N.A.
TP	mg/L	1.03 ± 0.04	N.A.
AN	mg/L	1.120 ± 0.053	N.A.
As	mg/L	N.D.	0.1
Cd	mg/L	0.001 ± 0.000043	0.01
Cr	mg/L	0.001 ± 0.000050	0.1
Cu	mg/L	N.D.	0.2
Fe	mg/L	0.035 ± 0.0017	0.1–1.5
Hg	mg/L	N.D.	N.A.
Mn	mg/L	0.004 ± 0.0002	0.1–1.5
Ni	mg/L	70.40 ± 3.46	0.2
Pb	mg/L	0.003 ± 0.0011	5
Zn	mg/L	0.005 ± 0.0023	2

*N.D., not detected; N.A., not available.*

### Plant Materials and Nickel Treatment

Healthy, certified seeds of *L. purpureus* (hyacinth bean) and *B. chinensis* (pak choi) provided by the Department of Agriculture, Penang, were applied as plant models. *L. purpureus* is a multifunctional legume, with a rich source of protein and a wide spectrum of therapeutic features. *B. chinensis* not only is a commonly consumed leafy cruciferous vegetables among Asian countries but also demonstrates an important value in Western diet (Sun et al., 2010), with its high resistance against different biotic and abiotic factors and a higher uptake coefficient for heavy metals. These crops have been subjected to the environmental risks of heavy metal-polluted soils and water by environmentalists (Gao et al., 2019; Wang et al., 2020).

The seeds of the plant models were surface sterilized with 75% (v/v) of ethanol, disinfected with sodium hypochlorite solution (2%), and rinsed thoroughly with deionized water. The seeds were cultivated in the hydroponic setups and constantly supplied with the aerated industrial wastewater effluent diluted at 0 (control), 5, 10, 25, 50, and 100%. The setup irrigated with the sole nutrient solution served as the control. The cultivation was conducted using 18 hydroponic setups in a greenhouse, with 16/8 h of photoperiod, 70–90% of relative humidity, and a mean day/night temperature of 30.6°C/23.9°C. The plants were harvested from the hydroponic growth substrate at the completion of the cultivation cycle and cleaned with deionized water to remove impurities from the adhering surface, before being stored for subsequent biochemical analyses.

### Bioaccumulative Potential

The plant samples were subjected to oven-drying at 80°C for 3 days before being ground to fine powder using a stainless grinder and digested by concentrated nitric acid in a microwave digester at 100°C. The heavy metal compositions of the plants was determined by ICP-MS.

### Growth Parameters

The growth rate was ascertained by the determination of the length of roots and shoots of the plant models. The length between the root–hypocotyl junction and the root tip was recorded as the root elongation, whereas the length from the plumule that emerged from the cotyledon to the farthest end of the leaf was taken as the shoot elongation. The growth rate measurements were conducted on a daily basis in triplicates.

### Chlorophyll Content

For the quantification of chlorophyll content, the aliquot (10 mg) of the ground leaves was immersed in acetone (80%, 3 ml) at 4°C, followed by centrifugation for 3 min (Heraeus Megafuge 8, Thermo Fisher Scientific, United States). The absorbance of the resulting extracts was recorded at the optimum wavelengths of 645, 470, and 663 nm (UV-1800, Shimadzu, Japan). The chlorophyll-*a*, chlorophyll-*b*, total chlorophyll, and carotenoid_(x__+__*c)*_ content [mg/g fresh weight (FW)] were determined with respect to the given equations, expressed by

(1) $$C\,h\,l\,o\,r\,o\,p\,h\,y\,l\,l-a=12.21\,A_{663}-2.81\,A_{645}$$

(2) $$C\,h\,l\,o\,r\,o\,p\,h\,y\,l\,l-b=20.13\,A_{645}-5.03\,A_{663}$$

(3) T⁢o⁢t⁢a⁢l⁢c⁢h⁢l⁢o⁢r⁢o⁢p⁢h⁢y⁢l⁢l=C⁢h⁢l⁢o⁢r⁢o⁢p⁢h⁢y⁢l⁢l-a+C⁢h⁢l⁢o⁢r⁢o⁢p⁢h⁢y⁢l⁢l-b

(4) $$C\,a\,r\,o\,t\,e\,n\,o\,i\,d\,s=\frac{1000\,{\text{A}}_{470}-3.27\,{\text{C}}_{\text{a}}-104\,{\text{C}}_{\text{b}}}{229}$$

where *A*_645_, *A*_663_, and *A*_470_ are referred to as the absorbance at 645, 663, and 470 nm, respectively, and the concentrations of chlorophyll-*a* and chlorophyll-*b* are represented by *C*_*a*_ and *C*_*b*_ (Lichtenhaler and Wellburn, 1983).

### Morphological Assessment

The changes of the morphological characteristics for the root, leaf, and shoot of the plant models were analyzed by using a scanning electron microscope (SEM). The plant specimens were prepared according to the procedure recommended by O’Brien and McCully (1981). The fresh root, leaf, and shoot samples were dissected from the same middle portion, and the obtained specimens, at a size of 5 mm^2^, were immersed immediately in 99% methanol for 20–40 s, followed by simple air-drying. The plant specimens were sputter-coated with gold, mounted on the aluminum stubs, and analyzed by 15-kV SEM (LEO Electron Microscope Inc., United States) (Pathan et al., 2010).

### Proline Level

The standard procedure outlined by Bates et al. (1973) was adopted for the quantitative calculation of free proline concentration. The homogenized filtrate of the fresh leaves was mixed with ninhydrin acid followed by acetic acid and heated at 100°C for 60 min. The resulting red organic layer was mixed vigorously after toluene extraction and applied for spectrophotometric determination of proline at the optimum wavelength of 520 nm, presented as μmol/g FW.

### Antioxidant Enzymes

The antioxidant enzymes of the plant models were extracted by homogenizing the plant material with a prechilled mortar and pestle in cold sodium phosphate buffer (pH 7.0, 50 mM) that contained ascorbic acid (0.20 mM) and PVP (1% w/v) (Jiang and Huang, 2001). After centrifugation for half an hour at 10,000 rpm (Heraeus Multifuge X1R, Thermo Fisher Scientific, United States), the resulting supernatants were withdrawn for the spectrophotometric analyses of antioxidative enzymes.

#### Guaiacol Peroxidase Assay

POD assay was initiated with the ambient incubation of the reaction mixture consisting of a supernatant (50 μl), guaiacol (100 μl), sodium phosphate buffer (3.75 ml), distilled water (50 μl), and H_2_O_2_ (100 μl) for 8 min. The absorbance of the mixture was recorded at 470 nm with reference to the reagent blank (Everse et al., 1994). The specific activity of POD was estimated by the specific extinction coefficient, *E* (26.6 mM^–1^ cm^–1^), and expressed as mmol/mg protein/min (Noctor et al., 2016).

#### Ascorbate Peroxidase Assay

The determination of APX activity was conducted by mixing 150 μl of supernatant with 0.25 ml of sodium phosphate buffer, ascorbic acid, and EDTA. H_2_O_2_ was added for the immediate initiation of the reaction, with reference to the absorbance value observed at 290 nm. The detoxification capacity of APX was computed, with an *E* value of 2.8 mM^–1^ cm^–1^ (Nakano and Asada, 1981).

#### Catalase Assay

The CAT scavenging activity, expressed in the form of required CAT concentration for the liberation of half of the peroxide oxygen, was estimated by mixing 200 μl of H_2_O_2_, 50 μl of the supernatant, 2.5 ml of potassium phosphate buffer, and 250 μl of distilled water, using an *E* value of 40 mM^–1^ cm^–1^ (Aebi, 1984).

### Protein Content

The Bradford (1976) method was adopted for the quantitative verification of protein content, by referring to the standard curve prepared from bovine serum albumin. The plant sample was subjected to homogenization in the mixture containing potassium phosphate buffer, 1 mM of EDTA, and 1% of PVP, which was subjected to centrifugation for 20 min (11,000 rpm, 4°C) (Heraeus Multifuge X1R, Thermo Fisher Scientific, United States). The resulting supernatant was mixed with 5 ml of Bradford’s reagent, and the absorbance was measured at 595 nm with reference to the reagent blank and presented as mg/g FW.

### Statistical Analysis

The effects of the industrial wastewater irrigation practice were analyzed using analysis of variance (ANOVA). Analysis was conducted with six biological replicates, and the significant difference between the control and the industrial wastewater effluent-irrigated groups was detected by Duncan’s multiple-range test. The correlations among the POD, APX, and CAT were ascertained using Pearson’s correlation test. All statistical analyses were performed using IBM SPSS version 24.0 at a significance level of *p* < 0.05. The significant differences between the tested groups were denoted using superscripted alphabets.

## Results and Discussion

### Heavy Metal Composition of Industrial Runoff and Metal Uptake Rate

The compositional characteristics of the nickel electroplating industrial runoff, together with the recommended limit for the irrigation requirement, are given in Table 1. The concentrations of the heavy metals in the electroplating industrial wastewater effluent ranged within the respective permissible limits for agricultural irrigation, with an extremely high nickel concentration at 70.4 ± 3.46 mg/L. The heavy metal uptake in the different parts of the plant models is presented in Supplementary Table 1. Generally, arsenic and mercury were not detected, while lower concentrations of cadmium, chromium, and lead were recorded, ranging from 0.008 to 0.011, 0.001 to 0.013, and 0.001 to 0.016 μg/g DW, respectively. Meanwhile, the concentrations of copper, manganese, iron, and zinc fell within the range of 0.001–0.025, 0.001– 0.027, 0.002–0.031, and 0.007–0.028 mg/g DW, respectively, with the highest concentrations detected in the roots of the plant models. Specifically, nickel was detected at the highest concentration ranging from 180.15 to 188.26 mg/g DW in roots, at 7.85–9.23 mg/g DW in shoots, and at 23.51–26.28 mg/g DW in leaves. These findings were well corroborated with the heavy metal uptake with wastewater irrigation as reported by Nzediegwu et al. (2019) and Ma et al. (2015).

In particular, the nickel uptake in plants is governed by the root systems via passive diffusion and active transport mechanisms (Seregin and Kozhevnikova, 2006). The ratio of the uptake between the active and passive transport may vary with the plant species, form, and concentration of nickel in the soil or nutrient solution (Dan et al., 2002). The soluble nickel ions could be transferred from the growing surrounding to the plant roots through the cation transport systems for copper, zinc, and magnesium ions and nickel–chelator complex, which involve the high-affinity nickel transport protein, metallothionein, and metallochaperones (Eitinger and Mandrand-Berthelot, 2000; Schor-Fumbarov et al., 2005). The translocation of nickel to the upper plant parts, including the shoots and leaves, is driven by the transpiration stream of the xylem, while the translocation to the meristematic parts of the plants, in particular young leaves, buds, fruits, and seeds, takes place in the phloem, with the regulation of metal–ligand complexes and protein binders (Amari et al., 2016). This uptake and translocation process has been reported to be affected by nickel concentrations, plant metabolism, acidity of soil or solution, or composition of other metals and organic matters (Chen et al., 2009). In the present work, *L. purpureus* and *B. chinensis* demonstrated the accumulation of nickel in the descending order of root > leave > shoot, parallel with the rising concentration of nickel electroplating industrial wastewater (Supplementary Table 2). Likewise, Chandrasekhar and Ray (2018) and Rizwan et al. (2017) reported a similar bioaccumulative behavior in *Eclipta prostrata* (L.) L and *Oryza sativa*. Exceedance of 50% of the nickel ions was retained in the roots, mainly due to the sequestration in the cation exchange sites of the xylem parenchyma cell wall and immobilization in the vacuoles of roots. Specifically, majority of these nickel ions were detected in the vascular cylinder, with less than 20% found in the cortex surface, indicating a high mobilization of nickel ions in the xylem and phloem (Sachan and Lal, 2017).

### Growth Retardation

**FIGURE 1:**
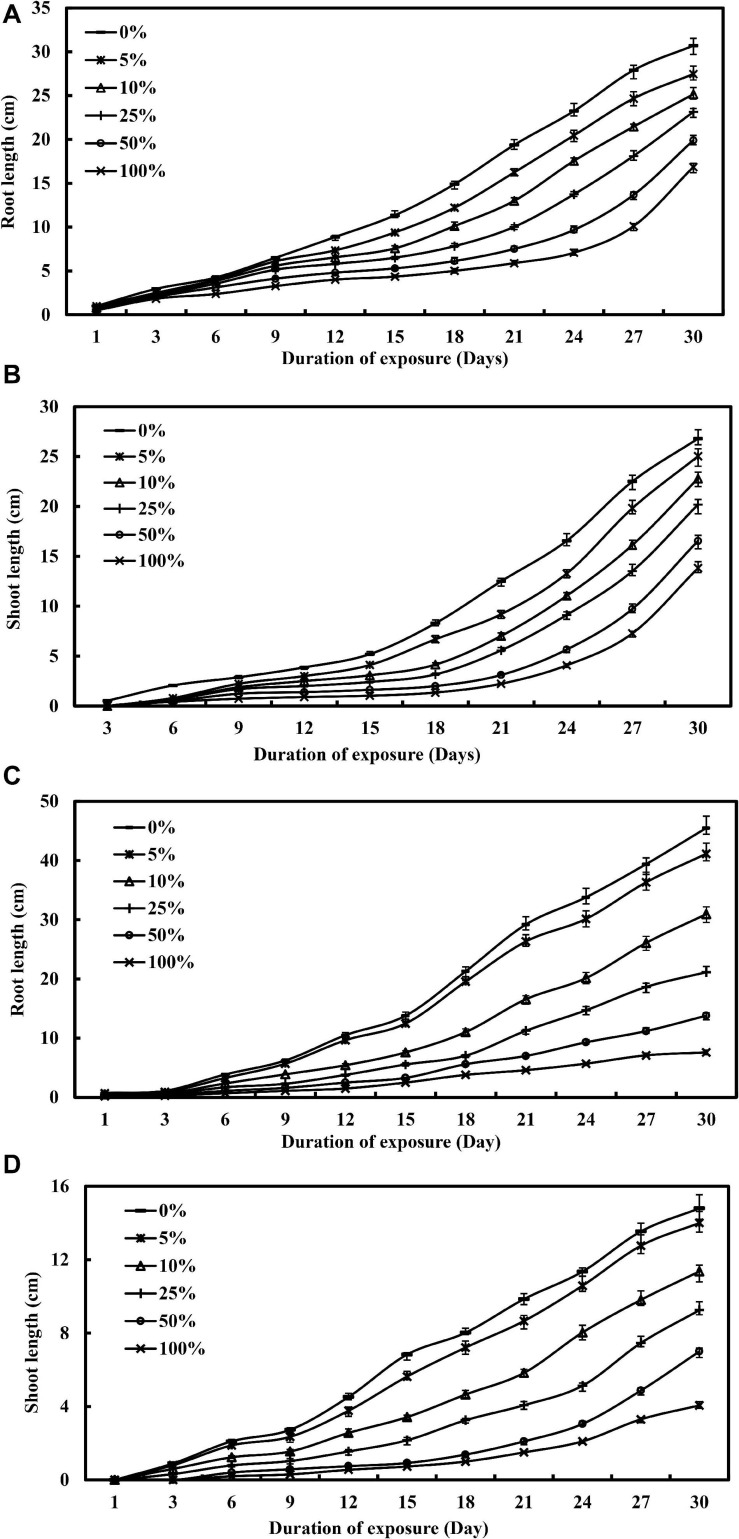
The visual toxicity of nickel electroplating industrial wastewater effluent for *Lablab purpureus* **(A,B)** and *Brassica chinensis* **(C,D)**, respectively, as a function of concentrations and duration of exposure.

Figure 1 illustrates the effect of industrial wastewater effluent irrigation practice on the lengths of roots and shoots of *L. purpureus* and *B. chinensis* as a function of concentration and duration of exposure. Increasing the concentration of wastewater effluent from 0 to 25% showed a gradual reduction in the root length of *L. purpureus*, from 30.7 to 23.1 cm (*F* = 132.5; *p* < 0.05), with the growth inhibition ranging from 10.6 to 24.6%. A dramatic root growth reduction was recorded beyond 25% of wastewater effluent, with an inhibition rate from 35.2 to 45.1% (*F* = 66.1; *p* < 0.05). The same concentration-dependent effect was apparently recorded for shoot length, with a growth reduction from 26.8 to 20.2 cm (*F* = 21.8; *p* < 0.05), while a profound reduction was observed beyond 25% of the industrial wastewater effluent-irrigated group. *B. chinensis* exhibited a minimal root growth reduction of 9.6% at 5% of wastewater effluent. Beyond this concentration, however, a drastic inhibition from 31.9 to 83.3% was observed (*F* = 165.2; *p* < 0.05). A similar but lower degree of reduction was recorded by the shoot of *B. chinensis*, with the percentage ranging at 5.4, 23.2, and 72.6% for 5, 10, and 100% of the wastewater effluent-irrigated groups, respectively (*F* = 133.5; *p* < 0.05).

A similar growth retardation was reported by Khan and Khan (2010), Parlak (2016), and Gupta et al. (2017) under nickel treatment within the concentration range from 0.01 to 40 mg/L, with a reduction from 1.4 to 97.8% for roots and from 0.4 to 89.4% for shoots in chickpea, pea plants, wheat, and millet. The growth impairment may be attributed to the cellular turgor loss, leading to the reduction of cell proliferation and cell division (Gabbrielli et al., 1990). The finding could also be ascribed to the intensification of the cell wall, driven by the lignification and polymerization reactions and regulated by the extracellular peroxidases, which governed the cross-linkage formation between the extension molecules and feruloylated polysaccharides. Additionally, nickel ions may significantly alter the fundamental metabolic pathways in plants, photosynthesis, and translocation processes to inhibit the normal growth of roots and shoots for different plant species (Shahzad et al., 2018).

### Alterations of Chlorophyll-*a*, Chlorophyll-*b*, Total Chlorophyll, and Carotenoid Content

**FIGURE 2:**
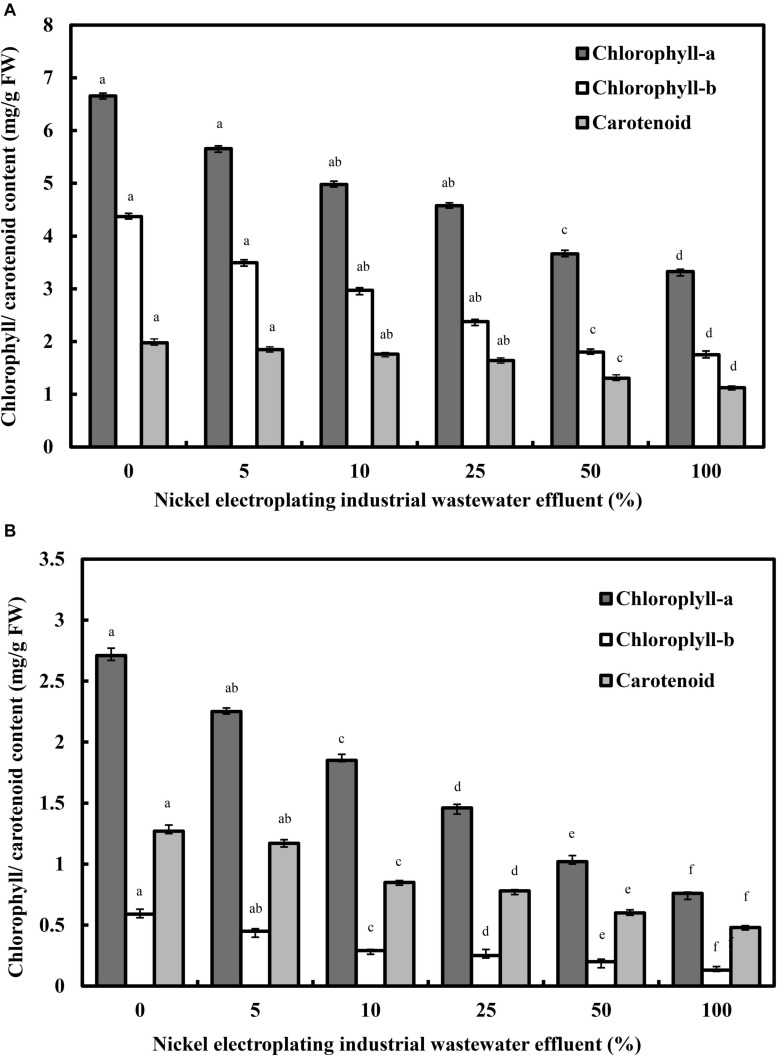
Alterations in photosynthetic pigments of *Lablab purpureus* **(A)** and *Brassica chinensis* **(B)**. The experiment was performed with six replicates, with different alphabets indicating significant difference.

The alterations of photosynthetic pigments in response to the percentage of nickel electroplating industrial wastewater effluent are illustrated in Figure 2. Chlorosis is considered to be a common toxicity symptom of nickel ions in plants. The rising nickel concentration in the irrigation water has induced a profound inhibition on the photosynthetic pigments of *L. purpureus*, with a statistically significant reduction from 15.2 to 50.4% for chlorophyll-*a* (*F* = 3,189.3; *p* < 0.05) and from 20.5 to 60.1% for chlorophyll-*b* (*F* = 1,900.9; *p* < 0.05). Similarly, increasing the percentage of wastewater effluent has caused a significant reduction of carotenoid content from 1.98 to 1.13 mg/g FW (*F* = 198.1; *p* < 0.05). Specifically, *L. purpureus* showed a drastic reduction in chlorophyll-*a*, chlorophyll-*b*, total chlorophyll, and carotenoid contents beyond 25% of industrial wastewater effluent. Comparatively, *B. chinensis* portrayed a higher vulnerability toward the rising percentage of wastewater effluent, with a dramatic reduction beyond 5% of wastewater effluent, ranging from 31.7 to 71.9%, 23.7 to 77.9%, and 33.1 to 62.2% for chlorophyll-*a*, chlorophyll-*b*, and carotenoid, respectively.

The phenomenon was mainly related to the deficiency of iron and magnesium or the substitutions of these essential ions by nickel ions at the tetrapyrrole ring of the chlorophyll molecules, leading to the denaturation of these chlorophyll structures (Küpper and Andresen, 2016). The chlorophyll reduction may be credited to the nickel-induced oxidative damage on the membranous structure of chloroplast (Fatemeh et al., 2012), with a drastic inhibitory impact on the biosynthesis of chlorophyll and degradation of functional photosynthetic pigments (Fuentes et al., 2014). Similar reductions have been found in the chlorophyll-*a* (46.5%), chlorophyll-*b* (39.5%), and carotenoid (52%) of nickel-treated spinach at the concentrations of 150 and 300 mg/kg of soil (Boostani et al., 2019), and a total chlorophyll reduction, from 34.5 to 43.9% and 39.2%, was detected in nickel-treated soybean at concentrations of 4 and 2 mM, respectively (Sirhindi et al., 2016; Mir et al., 2018). A specific point to be highlighted here is the higher ratio of chlorophyll-*a* to chlorophyll-*b* at 2.03 in *L. purpureus* after wastewater effluent irrigation, indicating a greater sensitivity of chlorophyll-*b* to nickel toxicity compared with chlorophyll-*a*. These acquired results verify the effect of nickel ion-induced oxidative damages on the membranous and molecular structures of chloroplast, with an inhibitory impact on the biosynthesis of chlorophyll, and degradation of functional photosynthetic pigments, to induce a dramatic impact on the Hill reaction, deteriorating the net photosynthesis and transpiration rates in the plant models as demonstrated in this work (Beri and Sharma, 2016).

### Morphological Changes

**FIGURE 3:**
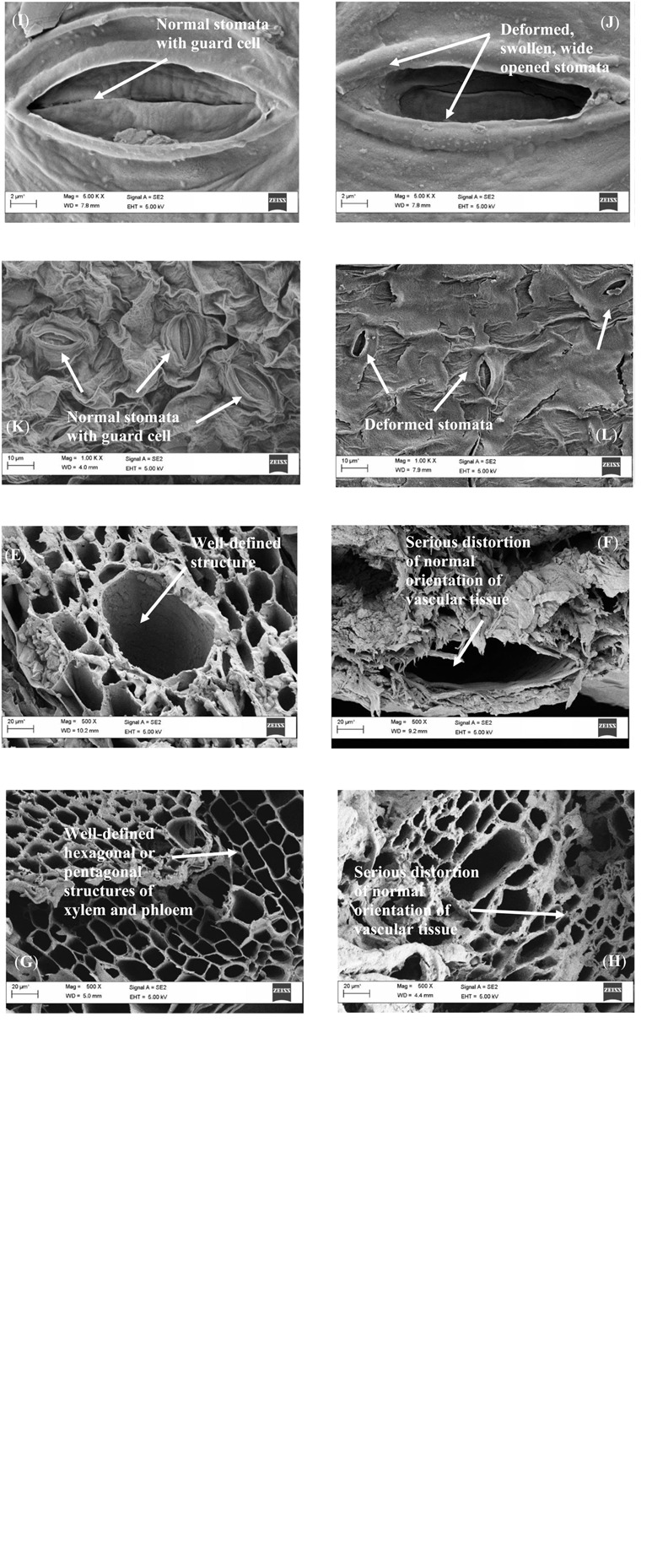
Scanning electron micrographs of the root surface for the control *Lablab purpureus* **(A)**, 50% wastewater effluent-irrigated *L. purpureus* **(B)**, control *Brassica chinensis* **(C)**, and 10% wastewater effluent-irrigated *B. chinensis* **(D)**, the transverse sections of the shoot of control *L. purpureus* **(E)**, 50% wastewater effluent-irrigated *L. purpureus* **(F)**, control *B. chinensis* **(G)**, and 10% wastewater effluent-irrigated *B. chinensis* **(H)**, the leaf of control *L. purpureus* **(I)**, 50% wastewater effluent-irrigated *L. purpureus* **(J)**, control *B. chinensis* **(K)**, and 10% wastewater effluent-irrigated *B. chinensis* **(L)**.

The representative alterations of the industrial wastewater-induced toxicity on the roots, shoots, and leaves of *L. purpureus* and *B. chinensis* are illustrated in Figure 3. From the presented images, the transverse section of *L. purpureus* root sample under the control condition demonstrated a normal stellar structure for both xylem and phloem tissues, to support the normal photo-assimilation process (Figure 3A). For the 50% of wastewater effluent-irrigated *L. purpureus*, the roots exhibited the structures of crimped xylem and phloem elements, representative of the impairment and disruption of vascular bundles, particularly the xylem vessels (Figure 3B). Well-developed structures of root xylem and phloem were portrayed by the control *B. chinensis* (Figure 3C), while 10% of wastewater effluent has seriously distorted the root tissue of *B. chinensis* (Figure 3D).

Similarly, the SEM findings of the transverse sections of *L. purpureus* and *B. chinensis* shoot samples revealed well-defined hexagonal or pentagonal structures of xylem and phloem (Figures 3E,G). However, 10 and 50% of wastewater effluent-induced irrigation resulted in serious deterioration on the orientation of these vascular tissues in *B. chinensis* and *L. purpureus* (Figures 3F,H). This suggested that the toxic effect of nickel ions has led to a subdued conduction of water and photosynthates.

The current findings highlighted the structural deformation of roots, with a noticeable alteration of the cell wall as compared with the control plant models, to induce a dramatic inhibition on the translocation or uptake of nutrient elements and water from the roots to shoots and the aboveground parts of the plant models. These results were in consonance with the findings reported in *Talinum triangulare* and *Vigna radiata* under lead- and mercury-induced toxicity (Kumar et al., 2013; Mondal et al., 2015). The structural malformation may also be attributed to the disintegration of the spongy parenchyma cells, resulting in a significant reduction of intercellular spaces.

From Figure 3I, the leaf samples of the control *L. purpureus* exhibited normal stomata with guard cells. Nonetheless, the exposure to 50% of wastewater effluent resulted in a deformed, swelling stomata, with the wide opening of stomatal apertures (Figure 3J). Similarly, structurally impaired stomata were observed in the leaves of *B. chinensis* under the irrigation of 10% wastewater effluent as compared with the normal stomata of the control (Figures 3K,L). Significant morphological alterations in the leaves not limited to the wide openings of stomata, with swollen surface, were consistent with the findings reported by Rai and Mehrotra (2008) in the leaves of the chromium-treated medicinal plant *Phyllanthus amarus* and by Mondal et al. (2013) in the leaves of cadmium-treated chickpea. This may be due to the preferential absorption of metal ions by subsidiary cells, and changes in the membrane permeability of the examined plant samples. The opening and closing of stomata were mainly governed by the alterations in the turgor and pressure of the guard cells, which resulted in the widening of the stomatal aperture. With the excessive accumulation of the metal ions, the swelling of intercellular substance between the guard cells took place, leading to the destruction of the connection between the cells and the guard cells, with the wide opening of stomata.

### Proline Content

**FIGURE 4:**
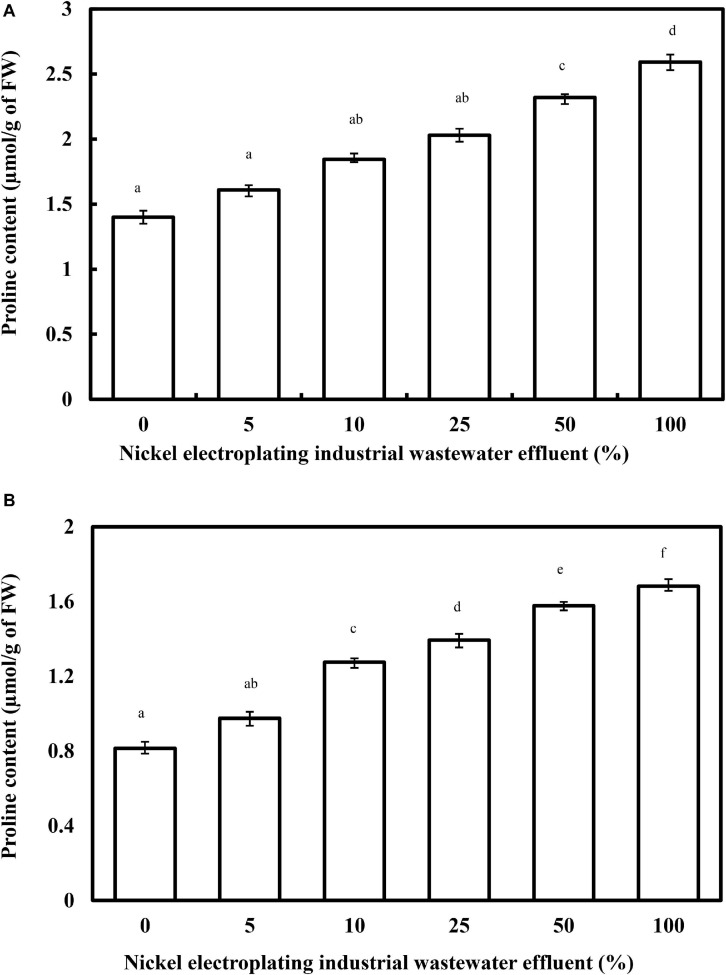
The changing proline content in *Lablab purpureus* **(A)** and *Brassica chinensis* **(B)** with respect to the rising percentage of nickel electroplating industrial wastewater effluent. The experiment was performed with six replicates, and different alphabets indicate significant difference.

As a multifunctional proteinogenic five-carbon amino acid, proline plays primary roles in protecting the plant system under metal stress, particularly for the stabilization of proteins, membranes, subcellular structures, osmoregulation, and ROS scavenging. Despite the non-fully-elucidated physiological significance of proline, the enhancement of proline content in different plant species has been well documented (Waseem et al., 2019). In this work, a profound concentration-dependent relationship related to the accumulation of proline content has been observed, with a progressive increase of proline content from 1.40 to 2.04 μmol/g FW by increasing the percentage of wastewater effluent concentration from 0 to 25% (*F* = 369.3; *p* < 0.05) (Figure 4). However, beyond the concentration of 25%, the proline content of *L. purpureus* exhibited a drastic upsurge from 65.6 to 85.3% (*F* = 313.5; *p* < 0.05). Parallel with the reductive effects in the physical growth and chlorophyll contents, the proline level in *B. chinensis* was increased from 19.8 to 106.6%, with a dramatic peak observed at 10% of the wastewater effluent.

El-Amier et al. (2019) have reported a similar phenomenon in the nickel-treated *Pisum sativum* that recorded 47.6% greater accumulation than the control group at a nickel concentration of 100 μM. The accumulative effect might be driven by *de novo* synthesis, lower degradation and utilization, or breakdown of proteins. The abrupt increase in proline accumulation beyond 5% in *B. chinensis* and 25% in *L. purpureus* could be ascribed to the nickel-induced toxicity, which has exceeded the maximum tolerable threshold level of these plant models and highlighted the higher vulnerability of *B. chinensis* toward nickel ions.

Accordingly, a number of metabolic mechanisms in the accumulation of proline against heavy metal-induced toxicity in plants have been elucidated. It is categorized into the adaptation, recovery, and signaling of stress tolerance. The accumulation not only emanated from the heavy metal-induced stress but also resulted from the water deficit syndrome (Kohli et al., 2019). In the present work, proline accumulation was recorded, which could be due to its leading function in osmoregulation or as an osmoprotectant, by adjusting the osmotic balance, altering the expression of specific genes to offset the water deficit syndrome, and controlling the stomatal closure to restrict the uptake or translocation of nickel ions via the suppression of transpiration (Hayat et al., 2012; Szepesi and Szõllõsi, 2018). The unique functional role of proline as a non-enzymatic antioxidant in the plant species under metal stress has been well established using both *in vitro* and *in vivo* studies (Kaul et al., 2008). Under metal stress conditions including nickel-induced stress, proline would prevent the inactivation of enzymes to upregulate the activities of peroxidases, superoxide dismutase, and CAT; induce the formation of phytochelatins; improve the rigidity of the cell wall; inhibit the lipid peroxidation reaction; and govern the potassium ion efflux across the membrane (Kaur and Asthir, 2015).

### Antioxidant Systems in Response to Nickel-Induced Toxicity

**FIGURE 5:**
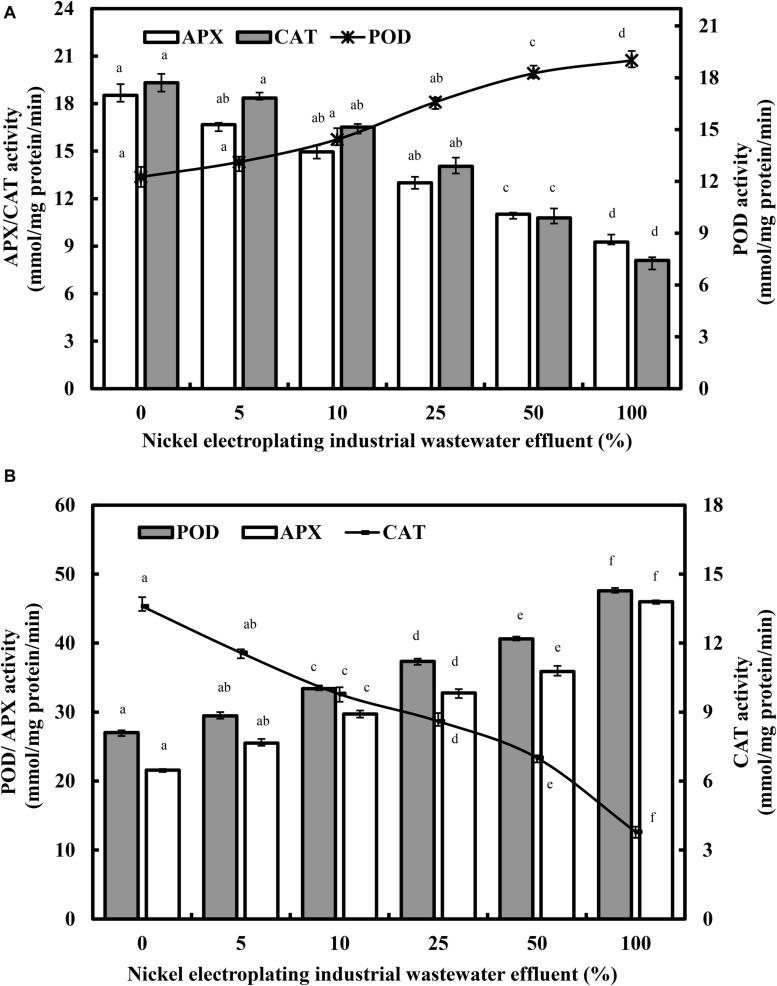
The changing activities of guaiacol peroxidase (POD), catalase (CAT), and ascorbate peroxidase (APX) in *Lablab purpureus* **(A)** and *Brassica chinensis* **(B)**. The experiment was performed with six replicates, and different alphabets indicate significant difference.

Figure 5 presents the varietal responses of POD, APX, and CAT enzymes in *L. purpureus* and *B. chinensis* subjected to nickel electroplating industrial wastewater effluent irrigation. Generally, the POD activities in both plant models were stimulated by nickel ions in a concentration-responsive manner. Increasing the industrial wastewater effluent from 0 to 100% promoted the rising POD activity from 12.23 to 18.90 mmol/mg of protein/min (*F* = 242.9; *p* < 0.05) in *L. purpureus* and from 27.02 to 47.60 mmol/mg of protein/min (*F* = 313.2; *p* < 0.05) in *B. chinensis*. Drastic upregulation of POD activities were observed beyond 25% in *L. purpureus* and 5% in *B. chinensis*.

Conversely, the APX activity was significantly inhibited from 18.52 to 9.26 mmol/mg of protein/min in *L. purpureus* (*F* = 289.5; *p* < 0.05), while *B. chinensis* demonstrated a concentration-dependent upregulation of APX activity from 21.58 to 46.01 mmol/mg of protein/min (*F* = 359.1; *p* < 0.05). However, in both plant models, CAT portrayed a similar trend of downregulated activity, from 19.32 to 8.10 mmol/mg of protein/min in *L. purpureus* (*F* = 436.4; *p* < 0.05) and from 13.60 to 3.79 mmol/mg of protein/min in *B. chinensis* (*F* = 156.9; *p* < 0.05).

**FIGURE 6:**
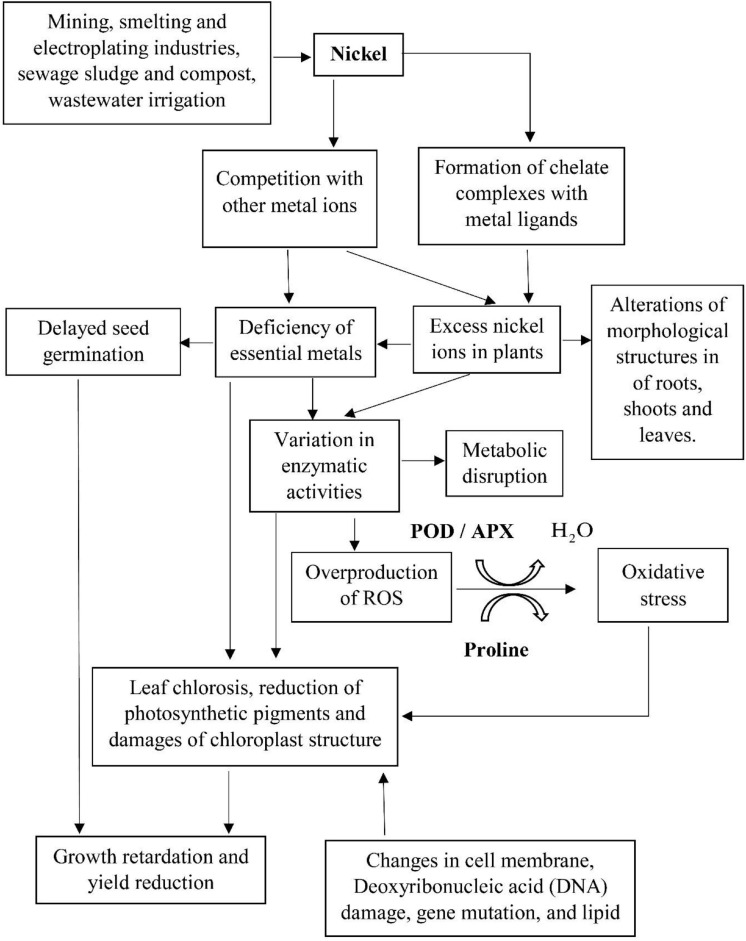
The primary sources of nickel ions, mechanism of nickel-induced phytotoxicity, and the associated antioxidant defense system against the damages on the macromolecules in plant cells.

The current findings on the concentration-dependent upregulation of POD agreed satisfactorily with those discovered in the nickel-treated groundnut and soybean (Gopal, 2014; Sirhindi et al., 2016). In the abiotic stress state, the main function of POD is the biosynthesis of lignin, hence the rising POD activity that may, in part, explain the hindrance of root and shoot growth in *L. purpureus* and *B. chinensis* as observed concomitantly in this study. Additionally, POD could be involved as a H_2_O_2_ scavenger in the lignification reaction in response to the nickel-induced phytotoxicity. Excessive nickel concentration may lead to the overproduction of ROS, with the interaction of different antioxidant enzymes (Figure 6). A low concentration of nickel exposure has been shown to enhance the POD activities with the activation of the antioxidant defense system (Gajewska and Sklodowska, 2005; Gajewska et al., 2006; Mir et al., 2018). Conversely, excess nickel ions have been found to be associated with lower ROS scavenging capability in plants, resulting in ROS accumulation and oxidative stress in plants. The abrupt upsurge of POD activity in *L. purpureus* and *B. chinensis* beyond 25 and 5% of irrigation water illustrated the threshold level of nickel-induced oxidative stress in the plant models, and a significantly higher POD activity is generally upregulated for ROS detoxification to protect plants from macromolecule damage.

On the other hand, the activities of CAT and APX were significantly suppressed in *L. purpureus*. The underlying mechanisms of the findings could be ascribed to the (i) reductive potential of nickel ions to induce iron deficiency of the APX metalloprotein complex in the plant tissues, as APX is a heme-dependent oxidoreductase whose catalytic activity is critically dependent on iron; (ii) binding of nickel ions with the thiol group of cysteine (Cys 32) at the active site of APX, leading to the inhibition of enzyme activity; and (iii) interactive reaction between nickel ions with the functional sulfhydryl groups, which might retard the enzymatic activity of APX in *L. purpureus* (Pandey and Sharma, 2002; Pandey et al., 2017; Karimi and Ghasempour, 2019). The activity of antioxidant enzymes may vary with the plant species, plant tissues, and stress duration, and this could justify the contrasting trends of APX activities in *L. purpureus* and *B. chinensis* (Gajewska and Skłodowska, 2007; Sreekanth et al., 2013). The rising activity of APX in *B. chinensis* could be an indication of the plant defense mechanism against free radical-induced toxicity, and the results were in concordance with those of the nickel-exposed *E. prostrata* (L.) (Chandrasekhar and Ray, 2018). The suppression in CAT activity was reported by Rao and Sresty (2000) and Gupta et al. (2017), which could be ascribed to the nickel-induced essential metal deficiency, resulting in the reduction of the biosynthesis of the iron porphyrin enzyme, as CAT is a metalloenzyme that contains Fe, Cu, Zn, or Mn in its prosthetic groups. Moreover, it has been demonstrated that the CAT activity was negatively related to the concentration of nickel ions in *Lycium barbarum* L. The low scavenging activity of CAT could be in part due to its presence in peroxisomes rather than in the chloroplasts or mitochondria, where majority of the radicals are generated (Pinto et al., 2019).

In the context of *L. purpureus*, the POD was negatively correlated with both APX (*r* = −0.942; *p* < 0.01) and CAT (*r* = −0.953; *p* < 0.01), while APX and CAT were positively correlated (*r* = −0.957; *p* < 0.01), indicating POD was the major antioxidant enzyme in detoxifying the excess-generated ROS under nickel-induced stress. Conversely, for *B. chinensis*, the positive correlation (*r* = 0.994; *p* < 0.01) between POD and APX, further verified by the negative correlations between CAT and POD (*r* = −0.977; *p* < 0.01) and APX (*r* = −0.988; *p* < 0.01), has supported the current findings that both of these antioxidant enzymes concurrently played concerted roles in the detoxification mechanism against nickel-induced oxidative stress. Differential antioxidant enzyme response toward the wastewater effluent irrigation indicated the factor of species variations toward nickel toxicity. The alterations of the POD, APX, and CAT activities under nickel excess in both plant models were in agreement with the literature findings, as presented in Supplementary Table 3.

Recent scientific research has highlighted the uptake and redistribution of nickel ions within the plant species via the pathway of cation and metal–ligand complex transport system during the exposure to nickel-contaminated water (Chen et al., 2009). The primary sources of nickel ions, the mechanism of nickel-induced phytotoxicity, and the associated antioxidant defense systems against the damages on the macromolecules in the plant cells are illustrated in Figure 6. In the present study, an excess of nickel ions has been depicted to exhibit toxic implications to *L. purpureus* and *B. chinensis*, verified by the inhibition of root and shoot elongation, diminution of chlorophyll content via the disruption of chloroplast structures, and alterations in morphological characteristics of roots, shoots, and leaves, and these perturbations in metabolic pathways could promote the overproduction of ROS. The augmented activities of POD and APX and accumulation of proline have been identified to be the key biomarkers of nickel-induced phytotoxicity to stimulate the defense machinery of *L. purpureus* and *B. chinensis* to accommodate the detoxification reaction.

## Conclusion

The present research has verified the viability of nickel electroplating industrial wastewater effluent diluted at different concentrations as a source of nutrient recycling using a hydroponic soilless cultivation system. The significant toxicity as evidenced by the inhibition of the root and shoot elongation and reduction of photosynthetic pigments, accompanied by the profound morphological distortions in the xylem, phloem, and stomata, was observed beyond the maximum tolerable concentration level at 25% of wastewater effluent for *L. purpureus* and 5% of wastewater effluent for *B. chinensis*. The accumulation of proline and upregulation of POD and APX activities were detected against the nickel electroplating industrial wastewater-induced oxidative stress injury in the plant models. Highlighting the different resistance of plant species toward nickel electroplating industrial wastewater toxicity, these findings contributed to a better understanding on the possible detrimental impacts resulting from the nickel-contaminated electroplating industrial wastewater irrigation on the food crops, which could be closely associated with the crop yield, plant physiological processes, and the sustainability of ecosystem via wastewater reuse.

## Data Availability Statement

The original contributions presented in the study are included in the article/Supplementary Material, further inquiries can be directed to the corresponding author/s.

## Author Contributions

YNC contributed to conceptualization, investigation, methodology, formal analysis, and writing – original draft. NAZ contributed to resources and funding acquisition. LKL contributed to investigation and supervision. KYF contributed to resources, conceptualization, writing – review and editing, visualization, supervision, funding acquisition, and project administration. All authors contributed to the article and approved the submitted version.

## Conflict of Interest

The authors declare that the research was conducted in the absence of any commercial or financial relationships that could be construed as a potential conflict of interest.

## Abbreviations

*a*: significance level probability of rejecting the null hypothesis when it is true
*A*_663_: absorbance at 663 nm
*A*_645_: absorbance at 645 nm
*A*_470_: absorbance at 470 nm
_(_*_*x*_*_+_*_*c*_*_)_: total carotenoids (xanthophylls and carotenes)
*C*_*a*_: chlorophyll-*a* concentration
*C*_*b*_: chlorophyll-*b* concentration
cm: centimeter
*F*: ratio of variation between sample means to variation within the samples
FW: fresh weight
mM: millimolar
*p*: probability value
*r*: Pearson correlation coefficient
*v*: volume.
