# Supplementary material for: Integrated Assessment of Nickel Electroplating Industrial Wastewater Effluent as a Renewable Resource of Irrigation Water Using a Hydroponic Cultivation System
Source: Front Plant Sci. 2021 Feb 23;12:609396. doi: 10.3389/fpls.2021.609396 (PMC7970764; doi:10.3389/fpls.2021.609396)
Supplement: Supplementary file 1 [file Data_Sheet_1.pdf]

Integrated assessment of nickel electroplating industrial wastewater effluent as a renewable resource of irrigation water using a hydroponic cultivation system

Y.N. Chow[1], L.K. Lee[2], N.A. Zakaria[1], K.Y. Foo[1*]

[1]River Engineering and Urban Drainage Research Centre (REDAC), Engineering Campus, Universiti Sains Malaysia, Seri Ampangan, Penang, Malaysia

[2]School of Industrial Technology, Universiti Sains Malaysia, Gelugor, Penang, Malaysia

*** Correspondence:**
Corresponding Author
k.y.foo@usm.my

Supplementary data

Table S1: Bioaccumulation of trace metal elements in the plant models grown.

Table S2: Nickel concentrations in different plant parts of *Lablab purpureus* and *Brassica chinensis* with the changing concentrations of nickel electroplating industrial wastewater effluent.

Table S3: A comparison of the guaiacol peroxidase (POD), catalase (CAT), and ascorbate peroxidase (APX) activities in nickel-exposed plants.

Table S1

Heavy metals	Unit	*Lablab purpureus*			*Brassica chinensis*		
		**Root**	**Shoot**	**Leaf**	**Root**	**Shoot**	**Leaf**
As	(μg/g DW)	N.D.	N.D.	N.D.	N.D.	N.D.	N.D.
Hg	(μg/g DW)	N.D.	N.D.	N.D.	N.D.	N.D.	N.D.
Cd	(μg/g DW)	0.011 ± 0.00040	N.D.	0.008 ± 0.00035	0.0088 ± 0.00035	N.D.	N.D.
Cr	(μg/g DW)	0.013 ± 0.0006	0.001 ± 0.00004	N.D.	0.009 ± 0.0003	N.D.	N.D.
Pb	(μg/g DW)	0.016 ± 0.0008	0.001 ± 0.00005	N.D.	0.015 ± 0.00066	N.D.	N.D.
Cu	(mg/g DW)	0.023 ± 0.0011	0.015 ± 0.0007	0.001 ± 0.00005	0.025 ± 0.0011	0.008 ± 0.0003	0.002 ± 0.0001
Mn	(mg/g DW)	0.025 ± 0.0011	0.002 ± 0.0001	0.001 ± 0.00005	0.027 ± 0.0012	0.002 ± 0.0001	0.002 ± 0.0001
Fe	(mg/g DW)	0.028 ± 0.0011	0.009 ± 0.0004	0.002 ± 0.0001	0.031 ± 0.0011	0.006 ± 0.0002	0.002 ± 0.0001
Ni	(mg/g DW)	180.15 ± 8.52	7.85 ± 0.28	23.51 ± 1.12	188.26 ± 8.82	9.23 ± 0.32	26.28 ± 1.11
Zn	(mg/g DW)	0.023 ± 0.0011	0.018 ± 0.00088	0.007 ± 0.00035	0.028 ± 0.0013	0.010 ± 0.00044	0.009 ± 0.00045

N.D., Not detected.

Table S2

**Concentration of wastewater effluent (%)**	**Nickel accumulation in *Lablab purpureus* (mg/g DW)**			**Nickel accumulation in *Brassica chinensis* (mg/g DW)**		
	**Root**	**Shoot**	**Leaf**	**Root**	**Shoot**	**Leaf**
**0**	N.D.	N.D.	N.D.	N.D.	N.D.	N.D.
**5**	7.64 ± 0.22	0.35 ± 0.016	1.12 ± 0.048	10.03 ± 0.48	0.40 ± 0.015	2.30 ± 0.08
**10**	38.02 ± 1.54	1.28 ± 0.054	5.65 ± 0.27	46.32 ± 2.08	2.53 ± 0.11	8.34 ± 0.35
**25**	55.31 ± 2.53	3.04 ± 0.14	11.83 ± 0.47	68.79 ± 3.21	4.86 ± 0.21	15.12 ± 0.73
**50**	100.37 ± 4.85	5.18 ± 0.23	17.67 ± 0.75	112.15 ± 5.52	7.10 ± 0.26	19.46 ± 0.76
**100**	180.15 ± 8.52	7.85 ± 0.28	23.51 ± 1.12	188.26 ± 8.82	9.23 ± 0.32	26.28 ± 1.11

N.D., Not detected.

Table S3

**Plants**	**Nickel ions concentration (mM)**	**POD (nmol/mg protein/min)**	**APX (nmol/mg protein/min)**	**CAT (nmol/mg protein/min)**	**Reference**
Lablab purpureus	0.06 - 1.20	12.26 - 19.00[a]	18.52 - 9.26[a]	19.32 - 8.10[a]	This study
Brassica chinensis		27.02 - 44.59[a]	21.58 - 46.00[a]	13.60 - 3.79[a]	
Cabbage	0.10	-	-	26.50 - 8.05	Pandey and Sharma (2002)
Pea	0.01 - 0.20	-	740 - 500[b]	69 - 65	Gajewska and Sklodowska (2005)
Wheat	0.10	1.80 - 6.70	-	-	Gajewska *et al*. (2006)
Groundnut	0.0001 - 0.40	0.94 - 1.96[c]	660 - 2150[b]	145 - 500	Gopal (2014)
Finger millet	0 - 0.68	0.55 - 1.25[d]		90 - 180[e]	Gupta *et al*. (2017)
Pearl millet		0.5 - 1.1[d]		80 - 180[e]	
Oat		0.35 - 0.85[d]		70 - 185[e]	

[a] mmol/mg protein/min; [b] nmol/mg protein; [c] Changes in A_{470} /mg protein; [d] U/g fresh weight; [e] mg H_2O_2 destroyed/5 minutes/g FW.
